# Structure and function: how to design integrated anatomy and physiology modules for the gross anatomy laboratory

**DOI:** 10.3389/fphys.2023.1250139

**Published:** 2023-08-08

**Authors:** Sara Allison, Caroline Mueller, Wendy Lackey-Cornelison

**Affiliations:** ^1^ Department of Biomedical Sciences, Western Michigan University Homer Stryker M.D. School of Medicine, Kalamazoo, MI, United States; ^2^ Department of Biomedical Sciences, Heritage College of Osteopathic Medicine, Ohio University, Dublin, OH, United States; ^3^ Department of Medical Education, University of Toledo College of Medicine and Life Sciences, Toledo, OH, United States

**Keywords:** gross anatomy, physiology, integration, laboratory, module, cognitive integration, backward design

## Abstract

Physicians must be able to integrate knowledge across disciplines. Therefore, educators need to provide opportunities for students to cognitively integrate information across the medical school curriculum. Literature has shown that specifically pointing out these connections helps students create cause and effect models and ultimately improve their performance. The gross anatomy laboratory provides an excellent environment for students to integrate information by establishing structure and function relationships. This article presents simple steps to create modules which help students cognitively integrate physiology and anatomy at the session level in the gross anatomy laboratory. Driven by backward design, these steps include establishing objectives, creating assessments, and developing activities that can be implemented in a specific learning environment. An example of a flexible module which could be implemented in a number of gross anatomy lab settings (e.g., prosection, dissection, models, virtual) is presented along with a template for the design of future modules. This is followed by a discussion of challenges encountered by educators attempting to integrate structure and function in the gross anatomy lab. Each of these considerations will be addressed with potential solutions for educators seeking to implement these types of integrated activities.

## 1 Introduction

Healthcare professionals frequently encounter complex problems that require integration of knowledge across disciplines. To meet this need, medical education programs must continue to identify opportunities for students to connect information across the many aspects of the curriculum. This is particularly true for the integration of anatomy and physiology as it is critical that students understand the relationships between structure and function in order to successfully address the multidisciplinary challenges they will face. Pedagogical literature has demonstrated that “knowledge organizations are most effective when they are well matched to the way that knowledge needs to be accessed and used” (Ambrose et al., 2010, p. 49). Additionally, the Liaison Committee on Medical Education (LCME) requires coherent and coordinated medical curricula that integrate content within and across academic periods of study (LCME, 2023). The Association of American Medical Colleges has also stated that student learning would be enhanced through integration of content (AAMC, 2001).

There are many approaches to curricular integration which can be used to integrate anatomy and physiology. Some of the most well-known methods of integration are horizontal, vertical, and spiral integration. Horizontal integration includes the integration of multiple disciplines related to a unified theme across a finite period of time (Brauer and Ferguson, 2015). In many medical schools, horizontal integration is accomplished by organizing basic science disciplines (e.g., anatomy, physiology, pharmacology, etc.) around a system (e.g., cardiovascular, gastrointestinal, etc.) during the preclerkship years. Vertical integration, on the other hand, is typically described as integration across broader time periods such as the preclerkship and clerkship years. In medical schools, this is often done by integrating clinical skills into the beginning of the program and foundational sciences into the later years of the program (Wijnen-Meijer et al., 2010). A spiral curriculum includes aspects of both horizontal and vertical integration by integrating across disciplines and time in a way that allows students to revisit topics in an increasingly complex manner as they progress through their education (Davis and Harden, 2003). To further categorize types of integrated teaching, Harden (2000) designed the integration ladder which describes 11 levels that range from isolation to complete integration: isolation, awareness, harmonization, nesting, temporal coordination, sharing, correlation, complementary, multi-disciplinary, interdisciplinary, and transdisciplinary. While the integration ladder has been widely utilized, others propose a simplified categorization structure of intradepartmental, interdepartmental, and consolidation (Sethi and Khan, 2019).

While there are many approaches to curricular integration, this alone is insufficient to prepare students to apply their knowledge to the multifaceted problems they will face as physicians. To be competent physicians, medical students must also achieve cognitive integration of basic and clinical science content. This type of integration goes beyond basic curricular design and involves a cognitive change in the learner’s mind when they make meaningful connections between topics (Kulasegaram et al., 2013). Educators often assume that curricular integration will inevitably lead to cognitive integration among students (Bandiera et al., 2018). They hope that when content from two different disciplines is presented in close proximity to each other, termed “temporal coordination” by Harden (2000), students will automatically develop the connection between the topics (Husain et al., 2020). But, Kulasegaram et al. (2015) demonstrated that this may not be the case. In their study, four groups of students were presented with explanations of brainstem strokes. The first simply received a list of clinical features of the brainstem stroke, the second received information regarding the anatomy and physiology of brainstem stroke followed by clinical features, the third received clinical features followed by the associated anatomy and physiology, and the fourth received an explanation which directly linked each of the anatomy and physiology points to a clinical feature. A week later, the fourth group that was given explicit connections performed significantly better on clinical vignettes related to brainstem strokes. Their study showed that proximity of topics is not enough and that cognitive integration requires explicitly exposing relationships between bits of information.

The gross anatomy lab provides an excellent opportunity to promote student’s cognitive integration of anatomy and physiology. In this environment, anatomical structures can be directly linked to physiological function. This integration provides meaningful context and relevance to the anatomical structures which ultimately helps make information easier to retain and recall (Woods et al., 2005). Adams and Dewsbury (2022) also found that a majority of undergraduate students preferred an integrated anatomy and physiology course over separate anatomy and physiology courses. Qualitative analysis revealed that students recognized that the integrated course would help them establish immediate connections between structure and function. They also perceived this method to be easier and felt it would lead to increased understanding.

Despite being an ideal setting for integration, the gross anatomy lab has remained somewhat isolated and there are limited studies that explore outcomes of integration in the gross anatomy lab. Some have designed professional development activities to help students develop scientific skills (Schön et al., 2022) and improve their reflective practice strategies (Lachman and Pawlina, 2006). Others have integrated clinical activities in the gross anatomy lab by including computed tomography (CT) scans of donors (Lufler et al., 2010); having pathologists assist students in determining the donor’s cause of death (Rae et al., 2017); and having students present a clinical condition the donor experienced during their life (Meredith et al., 2019). Drake (2007) created a clinical oriented anatomy lab which used cases to guide sessions in a prosection-based gross anatomy lab. Similarly, Mueller (2021) integrated clinical cases with guiding questions into medical student dissection manuals.

These important studies demonstrate the feasibility and benefits of integration in the anatomy lab; however, none have described a pedagogical approach to specifically help students cognitively integrate gross anatomy and physiology at the session level. In this perspective article, the authors utilize backward design to present step by step instructions to design modules which facilitate cognitive integration of anatomy and physiology concepts. These steps are followed by an example of a flexible module which can be implemented in a number of gross anatomy laboratory settings (e.g., prosection, dissection, models, virtual) and a discussion of solutions to potential challenges that educators face when attempting to integrate structure and function in the gross anatomy laboratory.

## 2 Steps to design an integrated module

To design laboratory modules which integrate physiology and gross anatomy, the authors recommend the following sequential steps supported by the backward design framework introduced by Wiggins and McTighe (2005): establishment of objectives, creation of assessment, and planning of the learning activity. In this section, the details of each of these steps will be discussed in the context of designing integrated modules that can be implemented in a variety of gross anatomy lab settings.

### 2.1 Establish the objectives

Backward design begins by establishing the desired results of a learning activity (Wiggins and McTighe, 2005; Wiggins and McTighe, 2012). By determining these learning objectives, educators can pinpoint what “knowledge, skills, or attitudes learners should be able to demonstrate following instruction” (Webb et al., 2013, p. 358). An effective learning objective requires several elements which describe who the audience is, what they will be able to do, how much of it will they be able to do, how well they should be able to do it, and by what time (Thomas, 2016). Beginning with this step benefits both educators and students. Specific objectives will guide the educator as they ensure alignment between the objectives, assessment, and learning activity. The objectives also serve as well-defined expectations that ensure transparency and guide students as they prepare for assessment.

During this initial step, educators should consider where in the curriculum an integrated module would be most appropriately placed. If the gross anatomy laboratories are situated within a systems-based curriculum, the educator may easily place modules which reinforce integration of anatomy and physiology throughout each of the systems. If gross anatomy is a stand-alone course organized by regional anatomy blocks, there may be specific regions that lend themselves to the creation of integrated modules. Once the educator has identified the appropriate placement of the modules, they can begin to pinpoint the key structure-function relationships and connections that students must know for their future practice.

At this time, it is also important to consider the level at which the students must understand the information and what they should be able to do with it. Objectives must be written intentionally to incorporate what type of cognitive skills students must utilize. Bloom’s Taxonomy (Bloom and Krathwohl, 1956) and The Revised Bloom’s Taxonomy (Krathwohl, 2002) are commonly used tools which help educators define levels of cognitive processes. Krathwohl (2002) builds on Bloom’s Taxonomy and includes six cognitive levels which increase in complexity: Remember, Understand, Apply, Analyze, Evaluate, and Create. These levels can then guide educators to select appropriate verbs for their objectives.

### 2.2 Create the assessment

Once the objectives are established, the next step is to create an assessment which verifies that students have met the objectives (Wiggins and McTighe, 2005). Ultimately, the carefully crafted objectives should guide the construction of the assessment but there are still a number of issues educators must consider. For example, educators need to decide if they will utilize a summative or formative type assessment. A summative assessment is understood by many as an assessment *of* learning while a formative assessment is regarded as assessment *for* learning (Taras, 2008).

Formative assessments are typically low stakes and serve to “provide feedback and correctives at each stage in the teaching-learning process” (Bloom, 1969, p. 48). Examples in the context of integrated anatomy and physiology modules may involve students briefly presenting conclusions drawn from the integrated module, taking a short oral post-quiz, or completing a multiple choice question quiz. Instructors may also want to include opportunities for students to demonstrate their knowledge on summative assessments. These assessments are often used to evaluate what a learner has achieved at the end of a course or program and can also come in a variety of formats from multiple choice exams and essays to portfolios and presentations. Whichever type of assessment is selected, it is critical that it aligns with the objectives and the level of objectives. For instance, if an objective is for students to evaluate a topic, it may be difficult to assess this using a multiple choice type question. Instead, an essay style question may be more appropriate and give students the opportunity to show that they have truly met the objectives.

### 2.3 Plan the learning activity

Following the design of the assessment, educators may now turn their focus to planning the activities that comprise the integrated anatomy and physiology module. The ultimate goal of the module is to ensure that students are able to achieve the specified objectives and this foundation should continue to guide the educator throughout their planning. Additionally, educators should consider a number of factors that will impact the specifics of their module.

Anatomy laboratories are taught through a variety of methods. Institutions may use prosection, dissection, models, virtual reality, augmented reality, computer-assisted learning, or any combination of these and other methods to aid students’ learning (Singh and Kharb, 2013; Duarte et al., 2020). The method used at the institution will play a large role in what the integrated module will be. Likewise, educators must consider how much time is available for the integrated module and at what point during the lab students could complete the activity. This will depend on how long the lab is and what other activities students are required to complete. Finally, educators must think about what types of resources and materials are available. The integrated module can be developed to utilize donors, models, dissectors, pre-lab materials, structure checklists, or any other resource that is available to the learners.

The challenge then lies in determining what type of integrated activity will best help students meet the objectives to cognitively integrate anatomy and physiology at the appropriate level while also working within the current structure of the lab. Depending on the context, this can include a variety of activities. Students can simply be asked to answer physiology questions related to tagged gross anatomy structures or match physiologic descriptions to gross anatomy structures. Modules could also include more complex activities such as team-based learning (Huitt et al., 2015), escape rooms (Molina-Torres et al., 2022), jigsaw activities (Crone and Portillo, 2013), or requiring students to create their own integrated questions for each other (Walsh et al., 2016). Educators can use elements of these established activities or create completely original modules. Regardless of the chosen methodology, the activity must align with objectives and assessment while also fostering students’ cognitive integration of anatomical and physiological concepts. This is done by centering the activity around opportunities for learners to explicitly link the two disciplines and create connections within their own mind (Kulasegaram et al., 2013).

### 2.4 A&P integrated module example

Table 1 illustrates an example of the design of a specific anatomy and physiology integrated module using backward design. This example module was developed for a dissection laboratory; however, this flexible module could easily be modified to work within a gross anatomy lab that utilizes prosection, models, or virtual anatomy. This template may also be used to guide the design of additional integrated modules.

**TABLE 1:** Integrated gross anatomy and physiology module example.

Step 1: Establish the objectives	
**Body Region/System** (e.g., cardiovascular, reproductive)	Gastrointestinal (GI) system
**Audience**	First year, first semester medical students
**Pre-Lab Knowledge Requirements**	Students will need to review basic GI anatomy (e.g., locations of specific organs) and physiology (e.g., cells and their function)
**Level of the Learner**	Beginner, recently introduced to material through pre-recorded videos
**Learning Objective(s)**	Correlate physiologic function with the anatomical structure of the stomach
**Revised Bloom’s Level**	Analyze

Step 2: Create the Assessment	
**Assessments** (formative, summative)	Formative—short oral quiz at the end of class
	Summative—multiple choice question on summative exam

Step 3: Plan the Learning Activity	
Part 1—Lab Structure	
**Instructional Methodology** (dissection, prosection, virtual anatomy)	Dissection lab
**Length of Lab Session**	2 h
**Lab Materials** (lab guides, prosection station, models, structure checklist)	Dissection instructions for stomach and celiac trunk dissection
	Dissection kits, gloves, etc.
	Pens, writing utensils
	Prosection of gastrointestinal system
Part 2—Creating the Activity	
**Describe the Activity**	Students will work in their assigned dissection table groups. They will work to complete the dissection of the stomach and celiac truck. Throughout their lab manual, they will have 4–5 related questions connecting the physiologic function to the anatomical structure. For example, when opening the stomach to view the rugae, three questions could be presented (answers provided):
	When food enters the stomach, the stomach stretches in response. Cells of the stomach respond to this stretching action by releasing gastrin.
	1. What anatomical feature of the stomach allows for the stretching?
	*- Rugae*
(worksheet that requires students to tag structures; case-based activity; escape room; students write integrated questions)	2. What is the name of the cells that release gastrin?
	- *G cells*
	3. What is the function of gastrin?
	- *Enhances gastric mucosal growth, gastric motility, and secretion of hydrochloric acid (HCl)*
**Time Needed for Activity**	5–15 min
**Which part of the lab is most feasible for integration?**	Throughout the dissection lab - students run through the questions on their own time, discussing the questions, researching the answers, etc.
(beginning or end of the session, entire duration)	
**Materials Needed for Activity**	Dissection instructions with interspersed physiology questions
(pins for tagging, models, prosected donors, worksheet, props for escape room, histological images)	Gastrointestinal models/prosections (optional)
	Atlases, textbooks, online resources

Step 4: Reflection on Activity	
**After the lab, reflect on the following questions:**	1. What worked well in the lab activity?
	2. What did not work well in the lab activity?
	3. Thoughts/comments on improvements?

## 3 Discussion of challenges and solutions

With increasing demands on faculty for scholarship and service, today’s educators often have limited capacity to create new content. The authors have identified common challenges that educators face when designing integrated modules for the gross anatomy lab. These primarily center around time, faculty, and course structure.

A major challenge facing almost every faculty member is a lack of time. Some may feel they simply do not have enough time to devote to the creation of an integrated module. In this case, the authors suggest starting small. Make a goal of designing just one module over the course of a year. This approach has the added benefit of allowing the educator to collect student feedback and make adjustments for the next module. Other faculty may face the challenge of very limited laboratory time. However, these integrated modules can take as little as 5 minutes for students to complete. They may even allow educators to cut material from other events and ultimately save time in the curriculum. Alternatively, the module could be completed after class as a review and self-study tool.

Implementation may also be hindered by faculty restrictions. Some educators may feel there are not enough faculty at their institution to facilitate these modules. In this case, the authors recommend printing answer keys or providing some other mode for learners to check their work on their own. This will allow students to work through the module independently while still getting the necessary feedback they need. Other educators may struggle with the process of module creation if they feel they do not have adequate discipline expertise. While this appears to be an obstacle, this scenario actually offers an excellent opportunity for collaboration. Seeking internal or external colleagues that have the content expertise will greatly improve the quality of the module.

Course structure may be another source of difficulty. Integrated modules as described in this article may seem difficult to implement in online or hybrid anatomy lab courses, particularly if these take place asynchronously. However, there are numerous resources for anatomical images (e.g., BlueLink^©^, Anatomy and Physiology Revealed^®^) that can be used to build modules using PowerPoint or electronic learning (e-learning) platforms. If necessary, these modules can be completed asynchronously and dialogue can be cultivated using a discussion board on a learning management system. Finally, if anatomy and physiology are taught in complete isolation it may feel nearly impossible to integrate the two disciplines in the anatomy lab. However, even if students enter the gross anatomy lab with no physiology background, there are still exercises which can introduce the connections between these two topics. This can be as simple as having students describe the morphological characteristics of a structure in the gross anatomy laboratory then use that information to justify its potential physiological function. This can lay the groundwork for further integration once students are exposed to both disciplines.

Each of these common challenges requires some flexibility and creative problem solving. These opportunities for learners to cognitively integrate structure and function are critical to their development and will benefit them far beyond the gross anatomy laboratory.

## Data Availability

The original contributions presented in the study are included in the article/Supplementary Material, further inquiries can be directed to the corresponding author.
